# Longitudinal Changes in Swiss Adolescent’s Mental Health Outcomes from before and during the COVID-19 Pandemic

**DOI:** 10.3390/ijerph182312734

**Published:** 2021-12-02

**Authors:** Beyhan Ertanir, Wassilis Kassis, Ariana Garrote

**Affiliations:** School of Education, University of Applied Sciences and Arts Northwestern Switzerland, 5210 Windisch, Switzerland; wassilis.kassis@fhnw.ch (W.K.); ariana.garrote@fhnw.ch (A.G.)

**Keywords:** COVID-19, mental health, depression, anxiety, stress, adolescence

## Abstract

This study aimed to explore changes in mental health outcomes (depression, anxiety, home, and school stress) from before the first COVID-19 wave (autumn 2019) to the later stages of the same wave (autumn 2020) in a sample of *N* = 377 Swiss adolescents (*M*_age_ = 12.67; 47% female). It also examined whether students’ background characteristics (gender, immigrant status, and socio-economic status) and reported COVID-19 burden predicted students’ outcomes and their intra-individual changes. Student’s mental health, background characteristics, and reported COVID-19 burden were assessed by a self-report questionnaire. The intra-individual changes in students’ scores were estimated using random coefficients regression analyses, with time points nested in individuals. To examine the effects of predictors (students’ background characteristics and the reported COVID-19 burden) on outcome scores and changes, multilevel intercepts-and-slopes-as-outcomes models were used. The results showed that the expected impact of the pandemic on mental health was not noticeable in the later stages of the first COVID-19 wave. Only two effects were demonstrated in terms of intra-individual changes, namely, an effect of gender on depression and anxiety symptoms and an effect of reported COVID-19 burden on school stress symptoms. Moreover, few associations were found for selected predictors and students’ mean level scores, averaged across both time points.

## 1. Introduction

The COVID-19 pandemic has caused enormous challenges with wide-reaching effects on the lives of young people, who are particularly affected by the pandemic. In June 2020, the United Nations Educational, Scientific and Cultural Organization (UNESCO; [1]) estimated that school closures affected about 900 million children and adolescents, or approximately half of the estimated global student population. Especially during the earlier stages of the first wave (March 2020–May 2020), families from all over the world were confronted with contact restrictions, distancing measures as well as closures of schools and leisure facilities (e.g., cinemas, theaters, and sporting arenas). Research on past epidemics that also involved quarantines demonstrated that restriction measures can be associated with deteriorations in mental health [2]. Moreover, the economy was in a global recession in 2020 [3], meaning more and more families were forced to face economic consequences through unemployment and a decrease in income. It was shown that economic pressure resulting from economic recession can favor family conflicts and tensions [4]. Using a sample of Greek adolescents, Motti-Stefanidi and Asendorpf [5] also confirmed that the Great Economic Recession had an impact on adolescents’ well-being and school adaptation outcomes. Thus, the extraordinary situation and consequences of the pandemic have the potential to change the conditions and familial contexts in which adolescents develop.

Moreover, adolescents need exercise and contact with their peers for healthy development. Schools and classroom settings are a crucial instance of socialization: through social relationships with peers, young people can form their own identity and begin to detach from the parental home [6,7]. In addition, adolescents face a range of developmental issues in their transition to adulthood [8]. They experience a variety of cognitive, social, and biological changes, which make adolescence a particularly sensitive and formative phase of life [9]. Therefore, in light of the COVID-19 research, it is crucial to examine the situation of adolescents.

One widespread concern is that individuals’ mental health has been negatively impacted by the COVID-19 pandemic [10,11,12] given that the increase in stressors (here: the pandemic) is expected to cause a decrease in psychological well-being and an increase in mental illnesses [13,14]. Immediately after the introduction of the shutdown measures, the World Health Organization [15] encouraged governments worldwide to consider their implications for the mental health of the whole population. Within a very short time, the number of scientific publications on the topic increased rapidly and reached its peak around the middle of the year in August 2020 [16].

Researchers assessing survey data from the earlier stages of the first COVID-19 wave have pointed to an apparent increase in the frequency and severity of symptoms of mental illness and distress [13,17,18]. However, the quality of most of the studies was restricted due to methodological limitations. The limitations concerned, for example, problems of representativeness, study design, or the general scientific quality of the publications. Particularly for the consequences of the first wave of the pandemic, only a small proportion of the published studies were peer-reviewed. More specifically, many research projects were implemented and published on preprint servers within a short time without going through journals’ regular quality-assurance processes [19]. Another drawback of the studies pertained to the study design. Most of the studies reporting higher levels of depression and anxiety related to COVID-19 impacts were cross-sectionally designed and mainly explored the short-term effects of the pandemic. In order to talk about pandemic-inducedchanges, studies must also rely on data collected before the pandemic [20]. In most studies, this was not the case.

In the meantime, more integrative research has been published showing that studies with several time point assessments within a longer time-period are scarce and that those that exist reported mixed findings [21,22]. For example, a meta-analysis of 25 longitudinal studies involving only adult samples showed that the psychological impact of COVID-19 lockdowns is small in extent and points to a more mixed picture [21]. The results confirmed the lockdown’s small yet significant negative effects on mental health (*g* = 0.15 for depression, *g* = 0.17 for anxiety), but failed to show significant effects on positive psychological functioning, suggesting that positive emotions remained unaffected. Similarly, another review and meta-analysis based on 65 longitudinal cohort studies verified significant initial increases in symptoms of mental illness; however, the initial rise was only moderate in magnitude [22]. Nevertheless, the results also indicated that the increase in mental health problems was particularly remarkable at the beginning of the pandemic (March–April 2020). For the subsequent time-period, a decrease in the reported incidence of mental health symptoms was observed, making the mid-2020 levels generally more comparable to those prior to the pandemic. Of the 65 studies included, only 10 studies were based on samples of adolescents, and some of those consisted of samples with pre-existing mental health problems [22].

Studies focusing on adolescents’ mental health in relation to the pandemic are scarce [23] and, to the best of our knowledge, there has not yet been a meta-analysis with an emphasis on adolescents. Nonetheless, a review by Jones et al. [23] and a paper analyzing the longitudinal changes in adolescent depression and anxiety symptoms from before and during the COVID-19 pandemic revealed that an increase in mental health problems has been observed in conjunction with the pandemic [24]. The results of the adolescent studies obtained from individual studies point to a heterogenous picture. For example, based on statistics from pre-pandemic studies, several cross-sectional studies from China reported higher incidence rates for anxiety and depression symptoms in children and/or adolescents than expected [25,26,27]. Additionally, a longitudinal study of 14- to 17-year-old US adolescents also found increases in symptoms of depression and anxiety [28]. Similarly, an Australian study confirmed an increase in the incidence of severe symptoms of depression and anxiety in a sample of adolescents aged 13 to 16 [29]. However, there are also studies showing no changes in symptoms of mental illnesses [30,31] or changes only in selected aspects of mental health. The results of a British study, for example, revealed longitudinal increases in depressive symptoms—but not in anxiety symptoms—in late childhood [32]. In sum, even though adolescence is a period of heightened vulnerability for the onset of internalizing psychopathology [33,34,35], the situation of adolescents during the pandemic in terms of mental health appears unclear and the inconsistent findings point to the fact that there is still limited evidence of longitudinal changes due to the COVID-19 pandemic [24].

### 1.1. Risk Factors Aggravating the Pandemic’s Effect on Participants’ Outcomes

The pandemic might not affect all individuals to the same extent. In the current COVID-19 literature, a number of risk factors aggravating the pandemic’s effect on participants’ outcomes can be found. Reported risk factors for mental health include female gender [36,37], low socio-economic status (SES) [17,18,20,38], and immigrant status [39]. Furthermore, different studies also proposed that the disease burden is a moderator on changes in mental health [24]. Empirical cross-sectional studies from China demonstrated, for example, that Chinese youth from Hubei, the hardest-hit region, had higher declines in psychological health than Chinese youth from other regions. Additionally, a prospective-longitudinal study from Switzerland also revealed that COVID-19-related stressors and coping strategies were associated with during-pandemic emotional distress in young people aged 22 [20]. More specifically, it showed that pre-COVID-19 emotional distress was the strongest predictor of during-pandemic emotional distress. Thus, it seems that individuals who already experienced high levels of distress in pre-pandemic times were particularly vulnerable to emotional distress during the pandemic. As a result, it is very likely that the sense of perceived burden can play a moderating role in terms of potential changes caused by the pandemic.

### 1.2. Present Study

On the basis of the aforementioned literature, in the present study, it is assumed that the COVID-19 pandemic and its containment measures have consequences on mental health. However, the existing research relies mainly on studies conducted in the earlier stages of the first wave of the pandemic. Those can only shed light on the short-term effects. Moreover, the extant literature’s quality is often restricted due to the quality of the measures used. For example, in terms of mental health issues, the common use of only selected, discrete items instead of internationally validated and reliable scales may have missed the psychometric properties that satisfied the criteria. Overall, it is apparent that there is still a lack of high-quality research exploring the later stages of the first wave of the COVID-19 pandemic, especially for the very sensitive period of adolescence. Against this background, the present study had three aims. First, using a longitudinal study with pre-pandemic data, we examined adolescents’ mental health during the later stages of the first wave of the COVID-19 pandemic and investigated whether and how the outcomes changed within one year. Second, we explored the effects of students’ characteristics on (a) the outcomes of mental health and (b) the changes in these outcomes. More specifically, we examined the predictive role of students’ gender, socio-economic status (SES), and immigrant status on the mental health outcomes, as well as on the intra-individual changes in these outcomes.

Moreover, even though the questionnaires of the current study did not include specific items related to the COVID-19 circumstances, answers to the open-ended question “What has bothered or burdened you significantly in the last six months?” indicated that a substantial proportion of adolescents considered the pandemic and its consequences to be definite burdening factors. That is why the data of the current study can be used to gain initial insights into potential midterm effects of the first wave of COVID-19 in terms of adolescents’ mental health outcomes. Thus, thirdly, this study also examined whether the reported COVID-19 burden predicted the observed changes in mental health.

### 1.3. Research Questions (RQ)

**RQ1** **(R1).** *Did the students’ mental health (depression, anxiety, home and school stress) change within the year that was shaped by the COVID-19 pandemic?*

**Hypothesis** **1** **(H1).** *It is hypothesized that students showed increases in symptoms of depression, anxiety, and stress during the first wave of the COVID-19 pandemic and its related containment measures.*

**RQ2** **(R2).** *Were the mean-level changes in students’ mental health predicted by students’ characteristics (gender, SES, immigrant status)?*

**Hypothesis** **2** **(H2).** *It is hypothesized that students who were Swiss nationals, male, and who had a higher SES experienced less dramatic increases in symptoms of depression, anxiety, and stress compared to female students, with immigrant status, and a lower SES.*

**RQ3** **(R3).** *Were the mean-level changes in students’ mental health predicted by the reported COVID-19 burden?*

**Hypothesis** **3** **(H3).** *It is hypothesized that changes in reported symptoms of depression, anxiety, and stress were moderated by the perceived COVID-19 burden. This means that burdened students experienced a greater increase in symptoms of mental illness and distress than students who did not report COVID-19 as a burden.*

## 2. Materials and Methods

The present study stems from the international “Overcoming Inequalities with Education” project, which was carried out in Switzerland, Germany, and Greece. The aim of the ongoing longitudinal study is to examine various aspects of school resilience in secondary school students from grades seven through nine with a special focus on students with immigrant backgrounds. According to the past research, immigrant background is considered as a risk factor for students’ resilience in education settings [40]. Therefore, in order to obtain a sample with a high proportion of immigrant students, the project targeted schools with a high percentage of students of immigrant background. Overall, 1070 students participated in the project across the three countries. However, as the data collection processes in Germany (attrition rate 70%) and Greece (attrition rate 31%) were disrupted by the second wave of the COVID-19 pandemic, only the Swiss data were used in the current study. Thus, the present study is based on *N* = 377 students for the first timepoint and *n* = 319 students for the second timepoint (attrition rate 15%). The data was collected in September/October 2019 (pre-pandemic data) and August/September 2020 (during-pandemic data). During the first wave of the COVID-19 pandemic, all schools in Switzerland were closed for eight weeks and officially switched to distance learning by 16 March 2020. However, on 11 May 2020, all schools reopened with almost all regular school operations taking place.

### 2.1. Participants

Twenty participating secondary schools were recruited in 2019 in three German-speaking Swiss cantons. All school principals expressing interest in the study subsequently identified classroom teachers willing to participate. We obtained signed parental consent forms for all participating students. All students who agreed to participate in the study filled out an online questionnaire in their classes on a regular school day. The total duration added up to approximately 45 minutes.

The mean age was 12.67 (*SD* = 0.68) for the first timepoint and the sample consisted of *n* = 210 (53%) boys. Of the students, *n* = 199 (55%) held Swiss nationality at the time of the first assessment and *n* = 163 (45%) indicated only other nationalities. The following countries were most commonly indicated as other or additional nationalities: Albania (*n* = 20), Bosnia and Herzegovina (*n* = 9), Germany (*n* = 27), Italy (*n* = 44), Kosovo (*n* = 22), Portugal (*n* = 11), Turkey (*n* = 36), and Serbia (*n* =10). In addition, 52 further countries were also named as study participants’ nationalities.

### 2.2. Measures

#### 2.2.1. Depression and Anxiety Symptoms

Depression and Anxiety Symptoms were assessed with the two subscales of the Hopkins Symptoms Checklist (HSCL-25; [41,42]). Fifteen items assessed symptoms of depression (e.g., “I feel melancholic”) and ten items assessed symptoms of anxiety (e.g., “I feel sudden fear without cause”). Responses could range from 1 = “not at all” to 5 = “very much”. Items were averaged to form two different subscale scores. Internal consistencies for the subscales in the first wave were α = 0.93 for the depression subscale (*M* = 1.79; *SD* = 0.67) and α = 0.86 for anxiety subscale (*M* = 1.95; *SD* = 0.62). The reliability scores in the second wave were also excellent, ranging from α = 0.94 for depression (*M* = 1.87; *SD* = 0.75) subscale and α = 0.87 for anxiety subscale (*M* = 1.95; *SD* = 0.64). Missing data on depression scores ranged from 5% at T1 to 17% at T2. Similarly, 4% of the anxiety scores at T1 and 16% at T2 were also missing for participants.

#### 2.2.2. Home and School Stress

The items of a shortened version of the Adolescent Stress Questionnaire (ASQ-S; [43]) were used to measure the stress experience of the adolescents. The original short form of the instrument consists of nine scales with different numbers of items ranging from two to four items per scale. However, the suggested nine-factor structure could not be proven in the German version [44]. That is why the current study included only items from the selected subscales *Stress of Home, School Performance,* and *School/Leisure Conflict*, referring to adolescents’ perceived level of stress concerning their home and school lives. A composite score was computed by aggregating the scores (summed across items) for each subscale (Home Stress vs. School Stress).

Respondents rated the level of stress they experienced in specific situations and during specific experiences on a scale ranging from 1 = “not stressful at all” to 5 = “highly stressful”. The internal consistencies of the subscales ranged from α = 0.80 (T1) to α = 0.84 (T2) for the Home Stress scale (T1: *M* = 2.60; *SD* = 1.14; T2: *M* = 2.77; *SD* = 1.17) and from α = 0.77 (T1) to α = 0.81 (T2) for the School Stress scale (T1: *M* = 2.72; *SD* = 0.78; T2: *M* = 2.72; *SD* = 0.84). Missing data ranged in home stress and school stress scores from 4 to 16%.

#### 2.2.3. Gender

Students’ gender was coded as 0 = “girl” and 1 = “boy”; the option “other” was not selected (5% missing data).

#### 2.2.4. Immigrant Status

The immigrant status was based on students’ self-reports concerning their nationality (0 = “Swiss”, 1 = “Other”). As is it legally possible to have several nationalities in Switzerland, two additional questions were also asked, namely, “Do you have any additional nationality?” and “If yes, please write in all your other nationalities.” Where students indicated Swiss nationality in the open question option, the immigrant status variable was adapted (4% missing data).

#### 2.2.5. Socio-Economic Status (SES)

Parental Education. Parental education was used as one of the proxies for students’ socioeconomic background. Participants were asked “What is your parents’ highest completed qualification or level of schooling?” Six response options, ranging from “she/he has not finished primary school” (= 1) to “university degree/higher education” (= 6), were presented for each parent separately (35% missing data). Based on these variables, we created one ordinal variable that indicated the highest level of formal education obtained by each participant’s parents, ranging from lower education to highest education (*M* = 4.14; *SD* = 1.01).

Access to a private room at home. A question in the questionnaire asked the adolescents whether they had access to a private room at home (6% missing data). The response options were “no” (= 0) and “yes” (= 1).

#### 2.2.6. Reported COVID-19 Burden

At both timepoints, we asked the adolescents to indicate their sources of burdens in the last six months with the question “What has bothered or burdened you significantly in the last six months?”. At the second timepoint, *n* = 255 of *N* = 377 students responded to the question. Thematic coding of their responses revealed three broad areas of burdens: school, COVID-19 and its consequences, and family. This thematic coding was used to quantify the burdens information. Based on this information, dichotomous variables/moderator variables were created. The thematic coding results indicated that categorizing the responses as reporting COVID-19 as a burden vs. not reporting any specific burden vs. reporting something other than COVID-19 as a burden made more sense instead of a single dichotomous moderator variable categorizing COVID-19 burdens vs. no burden/all other types of reported burden. Of the students, *n* = 86 indicated no burdens, *n* = 163 reported other types of burdens, and lastly, *n* = 64 students reported being burdened by the COVID-19 pandemic and its consequences (homeschooling and extraordinarily high proportions of exams in the period after the lockdown).

### 2.3. Analyses

The intra-individual changes in students’ scores were estimated using random coefficients regression analyses, with time points nested in individuals (models of step 1). The intra-individual slope in the outcomes were regressed on the binary time variable (0 = first timepoint (pre-pandemic score) and 1 = second timepoint (later stages of the first COVID-19 wave)) at the intra-individual level (within-level). Separate models were estimated for all outcome scores.

In order to examine the effects of predictors (students’ background characteristics and the reported COVID-19 burden) on outcome scores and changes, multilevel intercepts-and-slopes-as-outcomes models were used. For that purpose, we added the predictors in separate models as person-level (between-level) predictors and a cross-level moderator/interaction to the previously described models in multilevel intercepts-and-slopes-as-outcomes models. To investigate the effects of students’ characteristics (gender, SES, immigrant status), models of step 2 were estimated. In the next step, the moderator variable “COVID-19 burden” was added to the models as two dummy variables representing the different answer categories. The first moderator variable for the models of step 3 was the dummy variable (labeled as Burden A) for reporting COVID-19 as a burden vs. not reporting any specific burden (reference category). The second moderator variable (labeled as Burden B) for the models of step 4 represented the answer categories reporting something other than COVID-19 as a burden vs. reporting COVID-19 as a burden, which was the reference category.

All analyses were conducted with Mplus 8.4 [45]. The use of Full Information Maximum Likelihood Estimation (FIML) allowed us to handle missing data. Missing data (ranging from 3 to 35%) were due to response omissions or absence of participants at the data collection days.

## 3. Results

### 3.1. RQ1

To investigate whether the mental health scores changed within one year (RQ1), we regressed the scores on the dichotomous time variable at the intra-individual level (within-level) in multilevel random coefficients regression analyses. The results are displayed in Table 1.

The slopes show whether the mean-level scores increased or decreased from the first timepoint (pre-pandemic score) to the second timepoint (later stages of the first wave of COVID-19 pandemic). On average, only the adolescents’ depression and home stress scores showed a significant increase, and these changes were in the expected directions. However, there were no significant changes in the adolescents’ anxiety and school stress levels. Thus, our first hypothesis (H1) was only partially confirmed.

### 3.2. RQ2

To pursue our second research question (RQ2), we predicted the changes from the first timepoint to the second timepoint with random-intercepts-and-random-slopes-models by including students’ characteristics (gender, SES, and immigrant status) and controlling for age (see Table 2).

The inclusion of students’ characteristics revealed that the mean-level changes in depression and home stress scores were no more significant. This means that the increase in students’ depression and home stress levels were affected by students’ characteristics.

#### 3.2.1. Gender

In line with hypothesis 2 (H2), students’ gender predicted the mean score of depression, anxiety, and home stress levels, indicating that, on average, boys had lower depression, anxiety, and home stress scores than girls. Additionally, gender also predicted the intra-individual change in depression and anxiety. More specifically, girls had a significantly higher increase in depression and anxiety scores than boys. No significant effect of gender was visible for the changes in home stress, nor for changes in the stress of school life (both mean scores and changes). Thus, our H2 was only partially confirmed.

#### 3.2.2. Immigrant Status

Students’ immigrant status predicted the mean scores of depression symptoms and home stress, but not the mean scores of anxiety and school stress (see Table 2). The results showed that, on average, having an immigrant status was associated with lower home stress and higher depression scores. However, contrary to our expectations, immigrant status had no effects on the intra-individual changes of the scores. Thus, in general, our hypothesis regarding immigrant status and intra-individual changes was rejected.

#### 3.2.3. SES (Parental Education, Access to a Private Room at Home)

To explore the effects of SES, we estimated separate models including different two sub-indicators of SES. Except for mean anxiety scores and parental education, no significant effects of parental education were found for the mean mental health scores and changes thereof. Therefore, in the end, for the models of mental health outcomes, only the information regarding access to a private room was used as an SES indicator.

The models of the step 2 revealed that access to a private room predicted the mean scores of depression and anxiety. The negative significant estimates of the predictor variable indicated that, on average, students with access to a private room at home had significantly lower depression and anxiety scores compared to students who did not have access to a private room at home. No other mean scores or changes were significantly associated with access to a private room.

### 3.3. RQ3

In order to examine the effects of the reported COVID-19 burden (RQ3), we estimated different models with two types of dummy variables indicating the categories of burden. The end models are presented in Table 3.

The models of step 3 were run with the first dummy variable (burden A), including all students who reported COVID-19 as a burden vs. those not reporting any burden (reference category). An effect for the mean-level scores (intercepts) of mental health domains could not be found for the moderator variable of reporting COVID-19 as a burden vs. not reporting any burden. In terms of changes, the models of step 3 indicated that only the mean-level changes in school stress were significantly moderated by the reported COVID-19 burden, meaning reporting a COVID-19 burden was associated with higher increases in school stress when compared to students who did not report any burden. However, the mean-level changes in depression, anxiety, and home stress were not predicted by the “COVID-19 burden” moderator variable.

Next, we estimated separate models (models of step 4) with a second dummy variable (burden B), including all students who reported something other than COVID-19 as a burden vs. COVID-19 as a burden (reference category). Students who did not report any burden at all were not considered. These models yielded different results. In terms of home and school stress outcomes and changes, no significant effects of types of burden could be found. However, regarding the depression and anxiety outcomes, a significant effect of other types of burden was found. Students who reported burdens other than COVID-19 had, on average, significantly higher anxiety and depression scores (intercepts) compared to students who reported COVID-19 as a burdening factor. Moreover, students who were burdened by other factors also had significantly higher mean-level changes (slopes) in depression symptoms.

These results indicate that, except for school stress, reporting a COVID-19 burden was not a significant predictor of general changes in students’ mental health. On the contrary, other types of burdens seemed to have more impact on the adolescents’ mental health status. Hence, H3 was mostly rejected.

## 4. Discussion

The purpose of the study was to examine Swiss adolescents’ mental health outcomes during the later stages of the first COVID-19 wave in autumn 2020, and the potential impacts of the pandemic on students’ outcomes, by comparing data from before and during the first wave of the pandemic. In addition to exploring average effects, we also examined whether the inter-individual variance in the intra-individual trajectories was predicted by the students’ background characteristics (gender, immigrant status, and SES) and their reported COVID-19 burden. Overall, a general detrimental impact of the COVID-19 pandemic on all mental health domains was not found. However, the consideration of different predictor variables provided more detailed insight into the potential effects of the pandemic. The main findings can be summarized as follows:

First, without controlling for any students’ background characteristics, a significant increase in mental health outcomes could only be observed in the depression and home stress scores, but there were no significant changes in other mental health domains. Second, regarding the predictors for changes in students’ outcomes, the only effect we found was that of gender for depression and anxiety slopes. This means girls had a significantly higher increase in depression and anxiety scores than boys. Moreover, the immigrant status and SES indicators did not predict any changes in mental health scores. However, for some of the adolescents’ mean scores (e.g., students’ average depression scores across both time points), the effects of selected background characteristics were visible: gender predicted the mean scores for depression, anxiety, and home stress, meaning that girls had, on average, higher scores for depression, anxiety, and home stress, whereas immigrant status predicted only the mean scores for depression and home stress, indicating higher depression and lower home stress scores for non-Swiss students. Regarding the effects of SES, the results showed that a lack of access to a private room had a significant negative effect on students’ mean-levels of depression as well as anxiety scores. Thus, the SES indicator we used showed that low SES was associated only with higher depression mean scores, but not with any changes in the students’ outcomes.

Thirdly, the reported COVID-19 burden only predicted the intra-individual change in school stress, meaning burdened students had a significantly higher increase in perceived school stress compared to students who did not report any specific burdens. Moreover, the results revealed that students who reported other types of burdens had a significantly higher increase in depression scores compared to students who reported COVID-19 as a burdening factor, suggesting that other types of burden mattered more for the increase in depression scores rather than the reported COVID-19 burden.

Hence, these results indicate that the pandemic, as assessed in the later stages of the first COVID-19 wave, did not directly translate into a decline in mental health for students. Even without controlling for students’ background characteristics, the intra-individual changes in depression and home stress scores seemed to be within the expected ranges for this age span. Prior studies have demonstrated that increases in depression and stress levels are typical for early adolescence. Therefore, these increases may be due to developmental factors. For example, pre-pandemic studies reported an increase in depression of approximately *d* = 0.10 [33] to *d* = 0.25 [34] over time. In our study, the changes were within the expected range. Thus, with or without consideration of student’s background characteristics, there is no indication of an extraordinary increase or decrease in student’s outcomes as assessed in the later stages of the first COVID-19 wave. Consequently, our results regarding mental health symptoms align with the results of the studies [22,46] suggesting a normalization of mental health and the attainment of pre-pandemic levels in the later stages of the first wave.

One explanation for these results might be the fact that Switzerland’s containment measures were less strict, particularly in terms of school and leisure facilities, for children and early adolescents compared with those of its neighboring countries. Swiss schools for children of compulsory school age reopened on 11 May 2020, followed by all remaining educational institutions on 8 June 2020. At the same time, entertainment and leisure facilities such as zoos, sports grounds (especially for younger people), museums, and libraries also reopened, and restrictions on gatherings were loosened as well. So, after a strict eight-week lockdown and closures in the spring of 2020, normality returned to a great extent for the Swiss population. It is, therefore, very likely that the relaxed summer of 2020 could have had a compensatory effect, especially for the students’ mental health.

Our results also provide some hints in terms of potential risk factors. First, being a girl and/or having no access to a private room seem to be risk factors for mental health problems in early adolescence. It is a well-known fact that girls are more vulnerable than boys when it comes to mental-health-related concerns in early adolescence. Although our study cannot make statements about causal relationships between the pandemic and mental health, we can conclude that girls might be more at risk of developing mental health problems due to the pandemic than boys. Moreover, our results also point to the importance of access to a private room for their psychological well-being. Even though the moderating role of the SES indicator in terms of changes in mental health could not be confirmed, it can be assumed that having a private place to rest appears important for sustaining adolescents’ mental well-being. The finding that SES and immigrant status were not associated with increases in mental health problems contradicts some other studies expressing concerns that students with low SES might experience more psychological distress and stronger decreases in well-being due to the COVID-19 pandemic. One plausible explanation for these results could be Switzerland’s economic, social, and political stability, which continued during the pandemic under discussion: both the negative effects on families and the burdens acknowledged by adolescents were expected to be less than in many other countries.

### Limitations

Our findings should be considered within the context of several limitations. First, causality cannot be assumed from longitudinal, observational studies such as this one. Although no fundamental detrimental effects due to the pandemic could be observed in our study, it is important to note that a relatively long time had passed between the first and second assessment. Therefore, we cannot be certain that the associations reported here are caused by the pandemic. Second, the results relied on secondary analysis data that were originally collected with other research questions in mind. Hence, we did not include items that, in retrospect, would have been potentially useful for obtaining more detailed insights into the pandemic’s consequences. Moreover, our study also lacks assessment points during the first stages of the first wave of the pandemics, which might be useful for the understanding of the entire course of the first wave. Furthermore, some factors might have reduced the generalizability of the current findings. As Barendse et al. [25] reported, the strictness of government restrictions was a significant moderator of potential changes in adolescents’ mental health outcomes due to the pandemic. Thus, assuming that Switzerland had fewer pandemic-related restrictions than other countries, these results are not surprising. That is why the transferability and generalizability of our study results to other national contexts are limited. In addition, questions can be raised with regard to the operationalization of the moderator variables indicating the COVID-19 burden. Even though it is a strength of the study that the burden question was not a direct assessment of the COVID-19 burden—given that spontaneous COVID-19 burden reports can provide other useful information—we cannot totally exclude that students who did not report COVID-19 as a burden were not burdened by it. Thus, our results must be interpreted with caution. Lastly, our study does not cover the periods of later pandemic waves (i.e., the second and third wave in winter 2020/2021) and the renewed educational and social restrictions. Our results only provide insights into the situation in the end phase of the first wave in autumn 2020 and, thus, might only reflect the situation at that particular time. However, it is beyond the scope of the present paper to determine the short-term and long-term effects of the pandemic on adolescents’ outcomes. Further analyses and studies are needed in order to allow more detailed insights into the impacts of the pandemic.

## 5. Conclusions

Our results showed that the expected impact of COVID-19 pandemic and its related containment measures on mental health symptoms for adolescents in Switzerland was not noticeable in the later stages of the first COVID-19 wave. We assume that the initial increases in the number of adolescents reporting symptoms of mental illness noticed at the beginning of the pandemic may have not continued through the last stages of the first COVID-19 wave. Therefore, claims that COVID-19 pandemic containment policies have a dramatic effect on populations’ mental health seem to be unsupported by the current findings, at least for Switzerland in the specific time-period under examination. On the contrary, as also shown by the meta-analysis of Robinson et al. [22], our findings indicate that adolescents are mostly resilient to stay-at-home orders or other containment restrictions. However, even though the group of adolescents investigated here appears psychologically adapted to the challenges posed by the pandemic, our study also raises an important implication for educational administrators and public health professionals: in addition to counseling or other mental health support, access to other resources, such as private spaces for completing homework and spending time alone, can not only help to improve adolescents’ learning gains but also to strengthen their mental health.

## Figures and Tables

**Table 1:** Slopes of outcomes regressed on the binary measurement timepoint (models of first step).

	Intercept ^1^	Slope ^2^	*p*-Value for the Slope	Variance Outcomes (within-Level; Residual)	Variance Outcome Intercept (between-Level; Residual)	Variance Slope (between-Level; Residual)	ICC
Depression	1.828	**0.117**	**0.004**	0.188	0.293	0.107	0.517
Anxiety	1.950	−0.008	0.841	0.174	0.205	0.092	0.450
Home stress	2.672	**0.164**	**0.005**	0.334	0.910	0.385	0.601
School Stress	2.966	0.007	0.800	0.090	0.723	0.095	0.835
Reading Comprehension	21.281	**4.834**	**0.000**	25.044	93.644	2.563	0.745

Notes. *N* = 377; ^1^ = mean pre-pandemic score; ^2^ = change from T1 to T2 (later stages of first COVID-19 wave); ICC = Intraclass correlation. Significant estimates are in bold.

**Table 2:** Multilevel models (random-slopes-and-random-intercepts models) predicting the changes and mean scores of students’ outcomes.

	Depression	Anxiety	Home Stress	School Stress
	Step 2	Step 2	Step 2	Step 2
**Within-Level** *Regression weights*				
Intercept	2.121 (0.159) ***	2.308 (0.167) ***	2.825 (0.226) ***	3.277 (0.176) ***
Slope (change)	−0.167 (0.188)	−0.295 (0.765)	0.186 (0.246)	0.139 (0.108)
Between-Level Slopes (changes) on				
Gender (0 = male)	0.297 (0.081) ***	0.224 (0.076) **	0.101 (0.119)	0.085 (0.061)
Immigrant status (0 = Swiss)	−0.044 (0.076)	−0.086 (0.076)	−0.103 (0.118)	−0.044 (0.061)
Private room (0 = no)	0.171 (0.192)	0.236 (0.179)	−0.030 (0.250)	−0.174 (0.105)
**Between-Level** *Regression weights* Intercept on				
Gender (0 = male)	0.365 (0.067) ***	0.271 (0.059) ***	0.545 (0.114) ***	0.055 (0.096)
Immigrant status (0 = Swiss)	0.155 (0.067) *	0.057 (0.059)	−0.270 (0.117) *	−0.103 (0.098)
Private room (0 = no)	−0.576 (0.158) ***	−0.544 (0.166) ***	−0.294 (0.225)	−0.318 (0.180)
Variances				
Level 1 Variance	0.127 (0.361)	0.118 (0.207)	0.000 (0.000) ***	0.086 (0.385)
Intercept	0.250 (0.186)	0.191 (0.110)	0.938 (1.040) ***	0.635 (0.210) **
Slope	0.193 (0.725)	0.187 (0.417)	1.024 (0.099) ***	0.094 (0.770)
Covariance Intercept & Slope	0.066 (0.022) **	0.013 (0.020)	0. 031 (0.047)	0.058 (0.022) **

Notes. *N* = 377; estimates are unstandardized and significant estimates are in bold; standard errors are reported in parentheses; adolescents’ ages were included in all models as a covariate (grand mean centered). * *p* < 0.05. ** *p* < 0.01. *** *p* < 0.001.

**Table 3:** Multilevel models (random-slopes-and-random-intercepts models) predicting the students’ outcomes and the moderating effect of the reported COVID-19 burden.

	Depression		Anxiety		Home Stress		School Stress	
	Step 3	Step 4	Step 3	Step 4	Step 3	Step 4	Step 3	Step 4
**Within-Level** *Regression weights*								
Intercept	1.959 (0.221)	1.875 (0.190) ***	2.030 (0.163) **	2.177 (0.197) ***	2.899 (0.432) ***	2.546 (0.262) **	2.832 (0.171) ***	3.172 (0.216) ***
Slope (change)	−0.678 (0.241) **	−0.257 (0.224)	−0.979 (0.515)	−0.296 (0.240)	−0.049 (0.216)	0.306 (0.309)	0.256 (0.199)	0.253 (0.145)
**Between-Level** **Slopes (changes) on**								
Gender (0 = male)	0.125 (0.114)	0.338 (0.090) ***	0.102 (0.114)	0.228 (0.083) **	0.206 (0.175)	0.008 (0.132)	0.057 (0.095)	0.045 (0.070)
Immigrant status (0 = Swiss)	0.148 (0.112)	−0.154 (0.088)	0.061 (0.112)	−0.193 (0.086) *	0.129 (0.165)	−0.288 (0.137) *	0.019 (0.089)	−0.064 (0.072)
Private room (0 = no)	0.482 (0.247) *	0.170 (0.210)	0.804 (0.515)	0.186 (0.185)	−0.125 (0.222)	−0.010 (0.130)	−0.404 (0.202) *	−0.181 (0.119)
Burden A (0 = no Burden; 1 = COVID-19 burden)	0.090 (0.110)	--	0.033 (0.112)	--	0.089 (0.168)		0.173 (0.089) *	--
Burden B (0 = COVID-19 burden vs. 1 = other types of burden)	--	0.247 (0.104) *	--	0.178 (0.096)		0.024 (0.130)		−0.066 (0.082)
**Between-Level** **Intercept on**								
Gender (0 = male)	0.081 (0.084)	0.420 (0.075) ***	0.061 (0.073)	0.297 (0.069) ***	0.333 (0.189)	0.569 (0.133) ***	−0.227 (0.147)	0.105 (0.110)
Immigrant status (0 = Swiss)	0.184 (0.079) *	0.148 (0.080)	0.127 (0.068)	0.043 (0.070)	−0.332 (0.173)	−0.273 (0.138) *	−0.079 (0.139)	−0.162 (0.111)
Private room (0 = no)	−0.464 (0.223) *	−0.520 (0.179) **	−0.340 (0.164) *	−0.532 (0.191) **	−0.390 (0.424)	−0.095 (0.237)	0.135 (0.177)	−0.319 (0.196)
Burden (0 = no Burden; 1 = COVID-19 burden)	0.014 (0.079)		0.039 (0.68)		0.066 (0.177)	--	−0.004 (0.140)	--
Burden (0 = COVID-19 burden vs. 1 = other types of burden)		0.302 (0.079) ***	--	0.216 (0.069) **		0.179 (0.152)		0.198 (0.121)
Variances								
Level 1 Variance	0.116 (0.038) **	0.150 (0.337)	0.098 (0.027) ***	0.108 (0.231)	0.312 (0.058) ***	0.298 (0.035) ***	0.088 (0.545)	0.084 (0.451)
Intercept	0.165 (0.030) ***	0.239 (0.176)	0.119 (0.025) ***	0.203 (0.124)	0.807 (0.094) ***	0.781 (0.077) ***	0.584 (0.297) *	0.590 (0.244) *
Slope	0.226 (0.022) ***	0.111 (0.677)	0.239 (0.017) ***	0.153 (0.466)	0.362(0.044) ***	0.330 (0.028) ***	0.093 (1.089)	0.098 (0.902)
Covariance Intercept & Slope	0.015 (0.040)	0.034 (0.025)	0.004 (0.029)	−0.018 (0.022)	0.068 (0.072)	0.022 (0.054)	0.031 (0.032)	0.077 (0.025) **

Notes. *n* = 150 for models of step three, *n* = 227 for models of step four; estimates are unstandardized and significant estimates are in bold; standard errors are reported in parentheses; Students’ characteristics including age were included in models of step three and four as covariates. * *p* < 0.05. ** *p* < 0.01. *** *p* < 0.001.

## Data Availability

The datasets used in the current study are not publicly available, but parts of the data are available from the authors on reasonable request.
